# Model strategies to address barriers to cervical cancer treatment and palliative care among women in Zimbabwe: a public health approach

**DOI:** 10.1186/s12905-021-01322-4

**Published:** 2021-04-27

**Authors:** Oscar Tapera, Greta Dreyer, Anna Mary Nyakabau, Webster Kadzatsa, Babill Stray-Pedersen, Stephen James Heinrich Hendricks

**Affiliations:** 1 School of Health Systems and Public Health, University of Pretoria, Pretoria, South Africa; 2 Gynaecologic Oncology, Department of Obstetrics and Gynaecology, University of Pretoria, Pretoria, South Africa; 3 Radiotherapy Centre, Parirenyatwa Group of Hospitals, Harare, Zimbabwe; 4 Institute of Clinical Medicine, University in Oslo, Oslo, Norway; 5 Womens’ Clinic, Oslo University Hospital, Oslo, Norway; 6 Faculty of Health Sciences, Oral Health Hospital, Sefako Makgatho Health Sciences University, Pretoria, South Africa; 7 Faculty of Health Sciences, University of Fort Hare, East London, South Africa

**Keywords:** Cervical cancer, Zimbabwe, Barriers to access, Treatment, Palliative care, Strategies, Qualitative inquiry, Thematic analysis, Health policies

## Abstract

**Background:**

Cervical cancer treatment and care remains limited in Zimbabwe despite the growing burden of the disease among women. This study was aimed at investigating strategies to address barriers in accessing treatment and care by women with cervical cancer in Harare, Zimbabwe.

**Methods:**

A qualitative inquiry was conducted to generate evidence for this study. Eighty-four (84) participants were purposively selected for interviews and participation in focus group discussions. The participants were selected from cervical cancer patients, caregivers of cervical cancer patients, health workers involved in the care of cervical cancer patients as well as relevant policy makers in the Ministry of Health and Child Care. Participants were selected in such as a way as to ensure different of characteristics to obtain diverse perspectives about the issues under study. Discussion and interview guides were used as data collection tools and discussions/interviews were audio-recorded, transcribed and translated into English. Inductive thematic analysis was conducted using *Dedoose* software.

**Results:**

Salient sub-themes that emerged in the study at the individual patient level were: provision of free or subsidized services, provision of transport to treating health facilities and provision of accommodation to patients undergoing treatment. At the societal level, the sub-themes were: strengthening of health education in communities and training of health workers and community engagement. Salient sub-themes from the national health system level were: establishment of more screening and treatment health facilities, increasing the capacities of existing facilities, decentralization of some services, building of multidisciplinary teams of health workers, development and rolling out of standardized guidelines and reformation of Acquired Immunodeficiency Virus (AIDS) levy into a fund that would finance priority disease areas.

**Conclusion:**

This study revealed some noteworthy strategies to improve access to cervical cancer treatment and care in low-income settings. Improved domestic investments in health systems and reforming health policies underpinned on strong political are recommended.

**Supplementary Information:**

The online version contains supplementary material available at 10.1186/s12905-021-01322-4.

## Background

Cervical cancer is the fourth most common cancer among women worldwide and a recent GLOBOCAN 2018 report showed that 290,000 (51%) of the 570,000 new cervical cancer cases worldwide occurred in women living in low-middle income countries (500,000) (88%) [1]. Globally the average age of women at diagnosis of cervical cancer was 53 years ranging from 44 -68 years [2]. In Zimbabwe, cervical cancer is the most commonly diagnosed cancer among women and most cases are diagnosed at advanced stages. At least 2270 cases of cervical cancer are reported yearly, and 1451 deaths are caused by the disease annually across the country [3]. Despite the growing burden of the disease in the country, patients encounter a myriad of multi-dimensional barriers (e.g. lack of physical access to health facilities, limited services, lack of knowledge and high costs of services) in gaining access to screening, diagnosis, treatment and care [4, 5]. These challenges could be the underlying factors resulting in about 80% of the cases being presented in advanced stages [6].

With the global burden of cervical cancer disproportionately affecting women in low-income context, several barriers to treatment and care have been reported a review conducted by researchers in the United States of America. Inadequate infrastructure, limited access to preventive human papilloma virus (HPV) vaccines, screening, and treatment, as well as limited trained personnel and training opportunities continue to impede efforts to improve access to treatment and care in less developed world [7]. A similar review was conducted in Zimbabwe and it pointed out to similar challenges [8]. Health systems in low-income countries are overwhelmed with competing priorities and health care providers are often ill-prepared for the growing demand for their services. Inadequate resources, limited trainings opportunities and attrition of health workers are some of the challenges which overwhelm health workers in low-middle income countries [9]. A recent report from Latin America indicated that high cervical cancer incidence in that region was attributable to limited access to screening services and inadequate providers to perform diagnostic and therapeutic procedures. Geographical distances and cultural barriers were also cited as impediments to access and uptake of recommended diagnostic and treatment services for cervical cancer [10]. In South Africa, a recent study reported limited knowledge of cancer, lack of biomedical treatment and stigma as barriers to linkages to treatment and care by women with cervical cancer [11].

Zimbabwe has been experiencing a plethora of economic challenges that have resulted in weakening of social services including health care system [12, 13]. Most of the women at risk of cervical cancer are domiciled in rural areas (66%) and they live far away from the only two treatment centres in the country, located in Harare and Bulawayo cities [5, 8]. The country has about 105 cervical cancer screening health facilities, mostly located at the district level. The referral pathways for women suspected of cervical cancer is to provincial hospitals and then the two treating centres once cervical cancer diagnosis is confirmed using histological investigations available mostly in private laboratories. The treating centres operate day clinics during weekdays and while the services are subsidized, the costs are still beyond the reach of many. In addition, for chemotherapies, patients are required to buy their own drugs and related commodities such as needles and syringes. The available treatment options in the two treating hospitals are radiotherapy, chemotherapy and surgery. However, radiotherapy services are sometimes unavailable due to frequent breakdowns of machines [5, 8].

Several barriers to cancer treatment and care have been reported in Zimbabwe and these include: resource constraints, centralized diagnostic, treatment and palliative care services, shortages of specialists and high costs associated with treatment [14]. Our recent study demonstrated comprehensive multi-dimensional barriers to accessing treatment and care and these included: few treatment centers, lack of infrastructure, lack of commodities such as drugs, limited number of radiotherapy machines, frequent breakdowns of radiotherapy machines, high costs of services, few specialists, lack of standardized guidelines, limited health information system linked to cancers, lack of patient follow-up system, limited knowledge or inappropriate attitudes of health workers, frequent health worker strikes and bureaucratic referral system. Societal barriers reported were lack of knowledge, fear of being diagnosed of cervical cancer, stigma (defined in our context as segregation or discrimination associated with having cervical cancer), misconceptions (defined as having incorrect view or opinion due to lack of knowledge), family influences, attitudes and beliefs, influence of traditional and spiritual healers [5, 15]. Strategies suggested to fight against stigma in recent studies included involvement of traditional healers in interventions due to their community status [11, 16]. Kuguyo et al. [8] suggested increased funding for cervical cancer screening and community health education to promote early detection of the disease as some of the urgent interventions in Zimbabwe. To date most studies in low-income settings have focused on screening and secondary prevention of cervical cancer. However, this study was envisaged to unpack some of the key and contextual strategies which could be implemented to improve access and uptake of cervical cancer treatment and palliative care by women.

## Methods

**Fig. 1:**
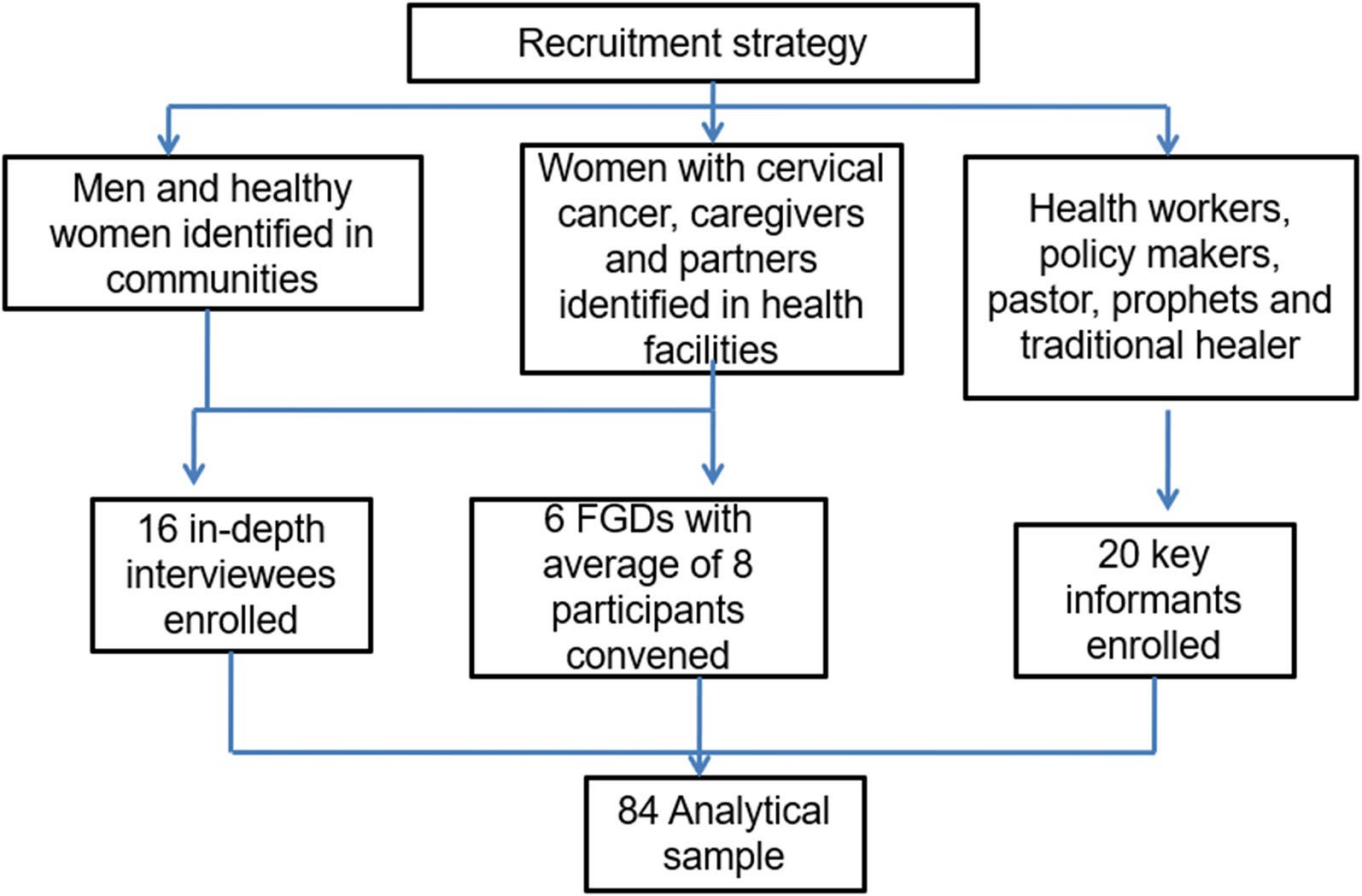
Flow diagram of participant recruitment for the qualitative study

This study was a qualitative inquiry conducted in Harare, Zimbabwe as part of a sequential explanatory mixed methods study. The main purpose of the study was to understand and explain some of the findings from the quantitative studies. The main relevant results obtained in the quantitative phase suggested significant access barriers to cervical cancer treatment and care among women which were not associated with socioeconomic status. In addition, cervical cancer treatment service supply was limited in the tertiary treating facility where this study was also conducted. The qualitative approach became useful in investigating some of the key strategies that could be implemented to address barriers to cervical cancer treatment and palliative care. A total of 84 participants were enrolled purposively in the study of which 16 were in-depth interviews, 20 were key informants (i.e. health workers, policy makers and spiritual leaders) and six focus groups with an average of eight participants each were conducted in different locations (see Fig. 1). All participants gave consent in writing before taking part in the study. The selection of the participants was conducted in the communities, health facilities and key institutions. Participants were selected based on knowledge and experience gained by interacting with women with cervical cancer or similar conditions. In-depth interview and focus group participants were identified during surveys in the communities and health facilities. Key informants were identified predominantly utilizing snowballing technique in which cases health workers in surveys would suggest names of potential participants to be considered for enrolment [17].

Figure 1 shows the flow diagram for the recruitment of participants for the qualitative study. A total of 84 participants were recruited for the study and these included 16 patients/caregivers, 20 key informants who comprised of health workers, policy makers and spiritual leaders and six focus groups of patients, caregivers and male partners.

### Data collection

All participants provided consent in writing to be interviewed and audio-recorded during the interviews and discussions. Interview and discussion guides designed by the researchers were used as data collection tools in this study [Additional Files 1, 2 and 3]. The main research question for this analysis was to understand some of the approaches which could be used improve access to cervical cancer treatment and care from different perspectives. The quantitative study had suggested significant barriers and limited service supply of cervical cancer services in Harare, Zimbabwe. The interview/discussion questions used in the data collection tools had been designed based on the findings from the surveys and the overall research questions. The researcher and his trained research assistant conducted the interviews and moderated the focus group discussions (FGDs) using the face-to-face approach. In-depth interviews and focus group discussion (FGDs) in communities were conducted in local churches and community centres. In addition to the audio-recordings, notes were also taken during each interview/discussion especially for observation of participants’ non-verbal communication. In-depth interview and FGD participants were given refreshments during the interview or discussion sessions in addition to an allowance of US$8 each to compensate for their time and travel [18, 19].

### Data analysis

Interviews and FGDs were transcribed verbatim and translated from Shona into English where applicable. Transcription and translation of audio-recordings were undertaken by the researcher and his assistant after receiving some training. Interview/discussion audio recordings were confidential and that the patients' personal information was handled appropriately at the end of the study to maintain privacy. Transcripts were identified by the unique identifier assigned (e.g. IDI01, KII01 or FGD01) to each participant (and stated by each participant at the beginning of the FGD/interview) rather than by any personal information. Unique identifiers were used to link the guides and the interview only after the conclusion of transcription. All in-depth interviews, key informant interviews and FGDs were coded manually line by line by the researcher using *Dedoose software* after creation of themes/sub-themes based on the research question and literature. Themes/sub-themes were discussed between the researcher and his assistant and only themes/sub-themes that were agreeable were considered. Manually generated thematic codes were processed in the same software to produce final outputs for the study. Coding is the process of highlighting text of interest and a code is text that is of interest to a researcher and is used to identify themes/sub-themes. The final themes were displayed as direct quotes [18, 19] and these were based on the research question.

## Results

### Main theme: strategies to address barriers to cervical cancer treatment and care

Most participants had high hopes that with enough investments and sound policies key barriers to accessing cervical cancer treatment and care could be removed. A myriad of sub-themes emerged related to some of the key strategies that could be implemented in the country to improve access and utilization of treatment and palliative care services by women with cervical cancer.

### Individual level sub-themes

#### Sub-theme: provision of free or subsidized services

Most participants reported that cervical cancer treatment and care was associated with high costs which could not be afforded by most people and hence the government should consider free or subsidized services. Some indicated that human immunodeficiency virus (HIV) and tuberculosis (TB) were being treated for free hence they felt that cervical cancer should also be considered for free treatment. Some respondents complained that the government and its partners were only providing them with free screening services yet if one is suspected of cervical cancer they would have to rely on out-of-pocket funding. One young caregiver suggested a strategy to assist cervical cancer patients to access treatment and care:

“@@@I think if the government could subsidize medication to allow people to get it at a cheaper price because someone from the rural areas who doesn’t work cannot afford the medication”. Caregiver, 20–30 years from Goromonzi.

One FGD participant mentioned that uptake for screening services was low because when one is suspected of cervical cancer they would need to pay high bills for treatment which they would not afford hence some women would opt not to be screened at all:

“@@@I don’t think it’s easy to go for screening because if you are suspected of cervical cancer the next stages needed for confirmation are very expensive so I think the government should lower the charges of cervical cancer servicesso that people can get treatment earlier”. Healthy woman, FGD participant from Hopely.

Another respondent reported that women with cervical cancer were dying due to lack of resources hence the government should do something to help them:

“@@@I think the government could facilitate for free treatment of cervical cancer for all women in Zimbabwe and it would alleviate the problem because many can actually die because of lack of resources to get treatment”. Cervical cancer survivor, 50–60 years from Shurugwi.

#### Sub-theme: provision of transport to treating health facilities through coupons or cash

Most respondents alluded to the centralization of cancer treatment services causing challenges for women who live in rural areas. The high costs of transport to treating facilities deter some women from seeking early treatment for cervical cancer. One respondent revealed that addressing the transport challenges would improve access and uptake of treatment and care services for cervical cancer.

“@@@Transport costs have to be small if people are to come in for treatment and care and if there are huge it has defeated our purpose. So that’s a very big policy issue that we need to discuss”. Key informant from World Health Organization (WHO) Country Office in Harare.

#### Sub-theme: provision of accommodation to women with cervical cancer during their treatment

With only two cancer treating health facilities in the country most respondents reported accommodation challenges during treatment sessions. Some of the patients have no relatives in major cities where treatment and care are provided hence, they are faced with many problems. One respondent explained her challenges in accessing treatment and care:

“@@@I do not have relatives in Harare so as a result my challenges will be much more and they include accommodation, food, transport and also medication”. Women with cervical cancer, FGD participant.

### Societal level sub-themes

#### Sub-theme: strengthening of health education in communities and training of health workers.

Some respondents reported that health education and cervical cancer awareness that were being given to communities were not adequate hence more was required in order to reach more people. Lack of knowledge about cervical cancer and its treatment was a source of misconceptions which were further impeding access and utilization of treatment services. One respondent revealed the limitations of the current awareness and health education interventions:

“@@@… I don’t think we are reaching enough in terms of education because we just give health education to those that would have visited the health centre and we don’t have outreach programmes that help us reach the communities.” Midwife, key informant from Harare Hospital.

Two other key informants also suggested strengthening of education for health workers:

“@@@…the health worker needs education in order to disseminate all the information, now they are disseminating the message that there is lots of cervical cancer and people should go and be screened and they stop there. Any other question asked they cannot go any further, so we need to educate and empower the lowest level of health workers who reaches the highest number of people in the communities”. Senior Pathologist, key informant from Parirenyatwa Hospital.

“@@@…..for health care professionals maybe our curriculum also needs to be adjusted to incorporate more cancer awareness as we go like what we have done with communicable diseases.” Pharmacologist, key informant from Harare.

An apostolic sect prophet proposed improved health education in communities to increase early detection of cervical cancer:

“@@@According to my thinking from the church side or prophecy the cancer care people should educate people in our churches”. Apostolic sect prophet, key informant from Zvimba.

#### Sub-theme: community engagement

Most respondents reported the need for community engagement in promoting uptake of screening and treating services for cervical cancer. Some of the community leaders that are crucial to engage because of their significant influence on communities include local leaders, traditional healers, pastors and prophets. One key informant had this to say:

“@@@…as Africans we know that you go to present whether to a village health worker or traditional healer or doctor or nurse when you are sick…” Senior Pathologist, key informant from Parirenyatwa Hospital.

A traditional healer reiterated the influence and importance of traditional healers in cervical cancer screening, treatment and care utilization:

“@@@…we [traditional healers] are seeing 80% of women with cervical cancer or the population in Zimbabwe consults traditional practitioners because they are accessible and their medicines are affordable”. Traditional healer, key informant from Harare.

Another community leader reported on their experience with women affected by cervical cancer:

“@@@…we have heard about it [cervical cancer] and we have been praying for people coming to church who had that challenge…..but we [pastors] are not well informed about the disease as there is no platform for the Ministry of Health to engage with the Church but we believe such a platform is essential for health related collaborations”. Senior pastor, key informant from Harare.

An apostolic sect prophet reported the importance collaborations with the government in order to improve access to cervical cancer treatment and care:

“@@@… the best is for people that have the knowledge on to come to centres like churches and ask to address the congregants because the moment you address maybe 1000 people gathered it means you have given information to almost 5000 people because the 1000 people will then spread the word out there…”. Apostolic sect prophet, key informant from Zvimba.

### National Health system sub-themes

#### Sub-theme: establishment of more screening and treatment health facilities

Most respondents suggested the establishment of more cervical cancer screening and treating health facilities across the country. This is due to the inadequate number of health facilities providing these services. A caregiver suggested the following strategy:

“@@@…if they could also increase the number of hospitals in different provinces that treat cancer of the cervix because some come from as far as Bulawayo and if you have no relative in Harare what will you do?” Caregiver,20–30 years old from Goromonzi.

One key informant reiterated the need to increase screening facilities through mobile clinics:

“@@@I think it can also go a long way if there are mobile screening services because remember some of our facilities patients need to walk some 5kms to access them.” Senior Gynaecologist, key informant from Harare Hospital.

#### Sub-theme: increasing capacity in screening and treating health facilities

Increasing the capacity of cervical cancer screening and treating health facilities was a salient emerging sub-theme especially among healthy women, women with cervical cancer and health workers. One health worker reported the following:

“@@@I think three broad areas: health education, capacity and costs are the major areas that we need to tackle, and others may come in as we go.” WHO Expert, key informant from WHO Country Office.

Another key informant reiterated the need to strengthen existing facilities to increase their capacities:

“…I think we need to improve the treatment centres that are available. Make them function optimally before we start thinking of establishing new centres.” Senior Oncologist, key informant from Parirenyatwa Hospital.

Another key issue that emerged is the issue of addressing persistent radiotherapy machine breakdowns to ensure uninterrupted services. One key informant reported the following with regards to the radiotherapy machines:

“@@@I think also the fact that access to treatment was not consistent in the sense that there were times when machines for treating patients were not working that also can be a negative push factor and needs to be addressed urgently”. Senior Oncologist, key informant from Parirenyatwa Hospital.

#### Sub-theme: decentralization of some services such as follow-ups

The salient strategy reported by most respondents was for the government to decentralize treatment and care of cervical cancer to lower levels of the health system across the country. A key informant reported the need to decentralize other services such as follow-ups of patients:

“@@@Concerning decentralizing some services such as follow up services I think that is imperative however; in terms of treatment I think we need to improve the treatment centres that are available. Make them function optimally before we start thinking of establishing other centres.” Senior Oncologist, key informant from Parirenyatwa Hospital.

#### Sub-theme: building of multidisciplinary teams of health workers in treating health facilities

Another sub-theme that emerged was the need to build multi-disciplinary teams of health workers to provide the comprehensive treatment services for cervical cancer. Teamwork cannot be underestimated given the multitude of care levels that cervical cancer patients have to go through as part of their treatment and care. Other registered practitioners such as traditional healers and prophets should also be engaged through their representative bodies in order to promote awareness and address stigma and misconceptions about cervical cancer:

“@@@We shouldn’t forget that we have other people who claim to know a lot about cancer in the communities and societies be they herbalist, traditionalists and spiritual healers that are saying things and you know somehow psychologically or traditionally black people tend to believe them” Senior Oncologist, key informant from Parirenyatwa Hospital.

The multi-disciplinary approach to cervical cancer treatment and care sub-theme was reinforced especially by oncology specialists:

“@@@What we are also lacking in terms of cancer care is that multi-disciplinary approach because then when you have multi-disciplines all coming together to work together at least for the good of the patient and at the end of the day you find that you will get reinforcement and collaboration of whatever needs to be done for the patient. If I may say even in this department, we don’t have social workers and psychologists, I mean we have oncology nurses, but they cannot double up to be everything.” Senior Oncologist, key informant from Parirenyatwa Hospital.

Another key informant explained palliative care and how it is provided by multiple health care workers using a team approach:

“@@@Palliative care is the treatment that is given to a patient from the point of diagnosis up to his or her death. It is also a multidisciplinary approach that involves the oncologists, physicists, radiographers, nurses, social welfare, family and church”. Oncology nurse, key informant from Parirenyatwa Hospital.

#### Sub-theme: development and rolling out standardized guidelines

Another sub-theme that emerged from the study was the issue of development and rolling out of referral and treatment guidelines to all levels of the health care system. One respondent explained the need for guidelines:

“@@@Yes people can say we refer a patient but I think we need guidelines such that if a patient is suspected of having cervical cancer they know that I’m going to go to this place to see either doctor A or doctor B or a cervical cancer clinic that they will just go straight into cervical cancer clinic without having to be booked into a general clinic because that helps”. Senior Oncologist, key informant from Parirenyatwa Hospital.

Another key informant reiterated the need for developing guidelines with input of multi-disciplinary team of health workers:

“@@@…we need to have guidelines that are set up by a multi-disciplinary team. It must be the whole team. It must be pathologists, it must be oncologists, it must be gynaecologists, it must be the nurses, epidemiologists coming together looking at the data, looking at the population coming up with guidelines that work for us here in Zimbabwe”. Senior Pathologist, key informant from Parirenyatwa Hospital.

#### Sub-theme: reformation of AIDS levy into a Fund that would fund priority disease areas such as cervical cancer

The AIDS levy was established to mobilize domestic resources to scale-up treatment and care for HIV/AIDS in Zimbabwe. However, HIV/AIDS is a known risk factor for cervical cancer yet the same fund does not cater for cervical cancer treatment services. Some respondents reported that the AIDS levy needed to be reformed so that it funds priority disease areas of which cervical cancer is one of them:

“@@@I think now there is so much to talk about cancer. The need is there people may feel overwhelmed if they are going to have another levy in addition to the AIDS levy but what I would recommend is that the AIDS levy can be slashed and then they share the AIDS levy and the Cancer levy but not to call it AIDS levy hoping that they will then share with cancer. There should be a separate budget for cancer”. Senior Oncologist, key informant from Parirenyatwa Hospital.

## Discussion

As the burden of cervical cancer is increasing particularly in low-income countries [1, 7], there are windows of opportunities for improving the existing health systems to increase access to treatment and care. This study revealed some of the strategies that could be implemented in Zimbabwe in the short to long term. At the individual patient level, this study suggests the following strategies: removal of user fees or subsidizing services, provision of transport to treating health facilities through coupons or cash and provision of accommodation to women with cervical cancer during their treatment. At the societal level the following interventions were suggested by our findings: strengthening of health education in communities and training of health workers and community engagement. Finally, at the national health system level our findings suggests the following interventions: establishment of more screening and treatment health facilities, increasing capacity in the existing screening and treatment facilities, decentralization of some services such as follow-ups, building of multidisciplinary teams of health workers in screening and treating health facilities, development and rolling out of standardized guidelines and reformation of AIDS levy into a fund that would finance priority disease areas including cervical cancer. While these strategies may not be exhaustive, they are a good starting point to drive cervical cancer treatment and care to priority in low-income contexts.

This study showed that removal of user fees or subsidizing treatment was imperative to remove the burden of high costs that impede linkages to treatment and care by women with cervical cancer. This finding is consistent with what was reported in the United States of America (USA), where Clinton Health Access Initiative and American Cancer Society found that cost was a major barrier to timely and quality care worldwide and they established a partnership in 2015 to optimize cancer drug market to improve access to affordable treatment [20]. Nyakabau et al. [14] recommended the availability of free or affordable and accessible chemotherapy in public health institutions to improve access and usage of treatment services. In a recent systematic review, researchers noted that fee exemption policies were one of the key strategies in improving access and utilization of maternal and neonatal care in sub-Saharan Africa [21]. Another study in Malawi revealed that user fees were a significant barrier to health care access and reduced detection of serious infectious diseases [21, 22]. These findings confirm our results which have demonstrated suboptimal access to cervical cancer treatment and care due to high costs.

Our study reported that challenges of transport to treating health facilities could be mitigated by interventions to provide transport assistance in the form cash or coupons to women with cervical cancer. However, this may be a short-to-medium term intervention in low-income settings. A USA study reported that patient transport or treatment centre accessibility could be improved through national commitment to infrastructural development such as road and rail networks, enhanced geographical distribution of treatment facilities and infrastructural support from non-governmental organization (NGOs) and industry [7]. This finding is long-term and more sustainable for settings with high incidences of cervical cancer however; such investments will take time and demand strong political will due to limited resources and competing priorities in low-income settings [5–7].

Cervical cancer treatment modalities usually involve women spending many days visiting treatment facilities for treatment whether radiation therapy or chemotherapy or a combination of different modalities. Unfortunately, at least 80% of cervical cancer patients in low-income contexts present late and they spend many days receiving treatment and care in tertiary health facilities [6]. In most cases accommodation challenges pose as a barrier to accessing and utilization of treatment and care services for patients who live outside major cities. One strategy to mitigate against these challenges is for the government and its partners to provide accommodation to cervical cancer patients during their treatment phases. Anecdotally, Tariro hostel at Harare Hospital used to accommodate cancer patients who were being treated at Parirenyatwa hospital until 2007 when it closed due to lack of resources. Re-capacitating the home to accommodate cancer patients from outside Harare would go a long way to ameliorate the challenges of accommodation and transport which were key emerging barriers to engagement into treatment and its adherence in this study.

This study revealed that strengthening of health education and training of health workers at all levels of the health system were imperative interventions to improve cervical cancer treatment access and usage. Lack of awareness has been reported as a barrier to cervical cancer screening uptake in low-income settings [23]. A South African study reported the importance of community outreach cancer education interventions involving traditional healers, churches and the youths to address stigma and other social ills associated with cervical cancer [11]. Researchers in the USA reported that collaboration of nations, international organizations and industry to develop and roll out workshops, courses and exchange programmes were key to improving health care knowledge and skills about cancer [7]. In Zimbabwe, researchers reported the importance of prioritizing training of primary care health workers in order to support the decentralization of some services to improve linkages to preventive and curative services [4]. Our work supports the findings and recommendations from the cited studies [6, 7, 11] and further reinforces the need for comprehensive community health education and health worker training interventions. Cervical cancer health education and awareness in not available in schools and tertiary learning institution in Zimbabwe and this could be established. Collaborative work involving government, academic institutions, international organizations and industry cannot be underestimated.

Findings of this study revealed the importance of community engagement through community leaders such as traditional or local leaders, pastors, prophets and traditional healers. These have significant influence in communities that could be used to promote uptake of preventive and curative services for cervical cancer. Given the complexities associated with cervical cancer as a disease and its treatment modalities community involvement is imperative [20]. Involvement of community leaders such as local chiefs, traditional healers, pastors and prophets is important to promote early detection and treatment of cervical cancer. Researchers in South Africa reported that anti-stigma interventions for cervical cancer could partner traditional healers as they have a critical role in society [11]. Our study supports this suggested intervention and further extends it to include other community leaders who command respect in African communities.

Increasing the number and optimally distribute cervical cancer screening and treatment facilities was a major salient sub-theme in our study. Another key sub-theme that emerged was the strengthening of capacities of the existing cervical cancer screening and treatment facilities to improve linkages into screening and treatment services. Recent studies have shown inequitable distribution of cervical cancer services and weak systems to provide comprehensive preventive and curative care services in low-income countries [6–8, 11, 20]. Capacity building for cervical cancer treatment and care would entail a list of different things from human resources, equipment, physical infrastructure, commodities, information systems, financial resources and leadership [24]. A Kenyan study reported that reliance on one radiotherapy machine by the whole country was a cause for poor health outcomes and higher cancer mortality rates [25]. Another study in the USA noted that patients with advanced gynaecological cancers wanted physicians to have a more active role in their psychosocial needs. Some of the needs identified were emotional, spiritual, patient-family communication, patient participation and decision making in advance directives [26]. These findings supported the results of our present study. However, despite the limitations and competing priorities, most low-income countries have strong political will and sub-regional, regional and international collaborations are potential mechanisms to build sustainable capacities [6].

Decentralization of some cervical cancer services was another key strategy reported in our study. Recently, researchers in Tanzania suggested that decentralization of service delivery for cervical cancer services was one of the key facilitators in initiating and expanding screening and treatment [22]. In Kenya in 2015, the government and its global partners established three more cancer treating centres in order to decentralize the diagnosis, treatment and care services to reach more people [23]. Nyakabau et al. [15] in Zimbabwe also proffered decentralization of some services as a policy priority in the nation to address barriers in the cancer continuum of care.

Findings indicate that building of multidisciplinary teams of health workers is strategic to improving access to high quality care and usage of treatment services. Taplin and colleagues [27] reported the effectiveness of multidisciplinary care teams (MDTs) in the cancer care continuum from screening to end-of-life. Multidisciplinary care teamwork improved follow-ups and adherence to prescribed procedure and reduced the time from diagnosis to treatment [27]. In Zimbabwe, this concept is still not in place due to several factors chief of which is limited number of specialists [5], however; there is need for the government and its partners to embrace some of these effective approaches.

Another key finding was that the development and roll out of guidelines for treatment and care of cervical cancer was an urgent priority across the country. The availability of guides and training resources for a complex condition such as cervical cancer cannot be underestimated on the background of poor health worker welfare which results in staff attrition in the country’s public health facilities which serves much of the populace [14, 15]. Alignment of the existing health infrastructure with cancer management is underpinned on the implementation of effective guidelines [5, 14]. It is within a sound policy framework that the government can effectively coordinate all activities to provide comprehensive cancer treatment and care.

A noteworthy finding from our research was the need for reforming the AIDS levy into a fund that would finance interventions for other priority conditions like cervical cancer. The AIDS levy was introduced in 1999 in order to mobilize resources to fight against HIV/AIDS in Zimbabwe [28]. The fund has performed well in terms of raising resources for HIV/AIDS intervention in addition to demonstrating commitment to fighting the disease which has also seen several global partners coming in to assist with more resources [29]. However, such a funding mechanism requires review considering other emerging diseases like cervical cancer. Treatment of cervical cancer is multimodal and complex hence it warrants the government to prioritize it given its high morbidity and mortality rates among women. Furthermore, donor funding for non-communicable disease has remained low over the past several years [29] signaling the need for low-income countries to come up with innovative strategies to raise domestic resources to finance non-communicable disease interventions. However; in low-to-middle income countries domestic funding for non-communicable diseases has been dominated by impoverishing out-of-pocket financing [28]. While introducing another levy on top of the existing one in Zimbabwe would be disastrous on the already overtaxed workforce, reviewing the AIDS levy to re-prioritize its beneficiary conditions may be a plausible starting point to raise domestic resources for priority conditions. Other innovative funding mechanisms such “sin” taxes levied on products like tobacco and alcohol may be introduced to complement the reformed AIDS levy. Collaborations between the government, international organizations and industry could also realize more innovative strategies to mobilize resources to improve cervical cancer treatment and care linkages in low-income settings.

Few studies, to our knowledge have investigated strategies that could be implemented to address broader barriers to cervical cancer treatment and care linkages in low-income settings. Future directions for cervical cancer control policy in low-income settings should consider: (1) sound and realistic policies underpinned by strong political will; (2) wider health system strengthening or diagonal approaches; (3) community engagement; and (4) collaborations with international organizations and industry to mobilize enough resources to finance interventions. Our study should be reviewed considering some limitations. Firstly, data was collected in Harare and therefore findings cannot be generalized to other contexts. Secondly, the study collected limited demographic information from participants in order to ensure confidentiality which the researcher had guaranteed during consenting process. Lastly, but not least some key policy makers in the Ministry of Health were not available for interviews hence the researcher had to work with those that were available. Despite encountering some limitations, this study had its fair share of strengths. The sample for this study comprised of diverse participants and these included: men, healthy women, women with cervical cancer, caregivers, managers from NGOs involved in cancer interventions, some policy makers from Ministry of Health, health workers, traditional healers, pastors and prophets. This culminated in a wider diversity of perspectives that enriched our outcomes. This study being qualitative, and a second phase of a sequential explanatory mixed methods design meant that issues were investigated very deeply to understand them better than would have been achieved in a quantitative design alone. Our study was further enriched using observations for non-verbal communication of participants which was crucial for understanding the subject under investigation. To the best our knowledge, this study was the first primary research in Zimbabwe to investigate the strategies that could be implemented to mitigate against barriers to accessing and utilizing cervical cancer treatment and care. Future policy directions could start from this work and other literature from similar contexts.

## Conclusion

In conclusion, our study advanced the scientific body of knowledge by demonstrating some of the key policy interventions that could be implemented to improve linkages to treatment and care by women with cervical cancer in a low-income setting. While the policy interventions identified are certainly not exhaustive, they are a good starting point to addressing challenges being faced by women in the country. Improved domestic investments in health systems and reforming health policies underpinned on strong political will are broader strategies that should be prioritized to sustainably reduce high cervical cancer morbidity and mortality in a low-income context.

## Supplementary Information

**Additional file 1**. In-depth interview guide.

**Additional file 2**. Key informant interview guide.

**Additional file 3**. Focus discussion guide.

## Data Availability

The datasets used and/or analyzed during the current study are available from the corresponding author on reasonable request.

## Abbreviations

AIDS: Acquired immunodeficiency syndrome
FGD: Focus group discussion
HIV: Human immunodeficiency syndrome
HPV: Human Papilloma Virus
NGO: Non-governmental organization
TB: Tuberculosis
WHO: World Health Organization
