# Supplementary material for: Model strategies to address barriers to cervical cancer treatment and palliative care among women in Zimbabwe: a public health approach
Source: BMC Womens Health. 2021 Apr 27;21:180. doi: 10.1186/s12905-021-01322-4 (PMC8077905; doi:10.1186/s12905-021-01322-4)
Supplement: Supplementary file 2 — Additional file 2. Key informant interview guide. [file 12905_2021_1322_MOESM2_ESM.docx]

**KEY INFORMANT INTERVIEW GUIDE**

| **DISCUSSION QUESTIONS** |
| --- |
| **Demographics of respondent**  • Can you please tell me more about yourself : Probe for: Age, ethnicity, education, occupation, place of residence, interaction with communities and how long they have been interacting with communities?  *Ndinokumbira kuti mundiudze zvizere nezvenyu. Bvunza makore ekuberekwa , rudzi, dzidzo yaakaita,Basa raanoita, nzvimbo yaanogara, , kushanda kwa anoita nevanhu vemunharaunda? Ane makore mangani achishanda nemnharaunda iyi?* |

| **Knowledge of cervical cancer**   - In your opinion do people know about cervical cancer? Probe for general knowledge, causes, signs and symptoms and risk factors.   *Munofunga kuti vanhu vanoziva nezve gomarara remuromo wechibereko?*  *Bvunza pamusoro peruzivo rwaka jairika, zvinokonzera, zvinoratidza and zvino nzwikwa nevanegomarara remuromo wechibereko , uye zvinonyanya kuita kuti munhu awane gomarara remuromo wechibereko.*   - According to your own understanding what do you think are some of the causes, signs and symptoms and risk factors of cervical cancer that you know?   *Mukuziva kwenyu zvii zvanoziva zvinoratidza nezvino nzwikwa nemhunu anegomarara remuromo wechibereko?*   - Can you tell me more about people’s awareness of preventing cervical cancer in your community?   *Mungandiudze zvizere pamusoro peruzivo rwekudzivirira gomarara remuromo wechibereko rune vanhu vemunharaunda yenyu ?*   - What are some of the measures that can be taken to prevent cervical cancer? Probe for screening and treatment of precancerous lesions, HPV vaccination and male circumcision of partners.   *Ndezvipi zvingaitwe mukudzivirira gomarara remuromo wechibereko? Bvunza pamusoro pekuongororwa nekurapwa kwezvironda zvinovapo gomarara risati ratanga, kubayiwa mishonga yekudzivirira ne kuchecheudzwa kwevarume*   - What are your opinions of your community with regards to risk of developing this disease and why do you say so?   *Mungandiudze zvamunofunga pamusoro pekuti vanhu vemunharaunda yenyu varipanjodzi yekuva ne gomarara remuromo wechibereko? Uye sei muchifunga kudaro?* |
| --- |
| **Experiences of cervical cancer**   - What have been your experiences with cervical cancer survivors?   *Ndezvipi zvamakasangana nazvo mukushana nevanhu vakararama shure mekuva ne gomarara remuromo wechibereko*?   - What are some of the reasons why women go for cervical cancer screening? Probe for routine screening, health problems, health worker advice, friend or relative advice?   *Ndezvipi zvimwe zvikonzero zvinoita kuti vanhukadzi vaende kuno ongororwa gomarara remuromo wechibereko?Bvunza pamusoro pekuenda kuno ongororwa nguva ne nguva, matambudziko ehutano, kukurudzirwa nevanoshanda kuzvupatara, ne shamwari kana hama?*   - What are your opinions on the awareness of treatment and palliative services for cervical cancer in your community?   *Munofunga kuti vanhu vamunharaunda yenyu vanoziva nezve kurapwa nerutsigirwo runopiwa neavo vanochengeta vanegomarara remuromo wechibereko?*   - Tell me more about your knowledge of cervical cancer treatment and palliative care services in Harare? Probe for where the services are offered.   *Mungandi udze zvizere pamusoro pe zvamunozviva pamusoro pekurapwa gomarara remuromo wechibereko kana kuno wanikwa rubatsiro kune vanochengeta vane gomarara mu Harare? Bvunza kuti rubatsiro runopiwa kupi?*   - Can you tell me what you know about support from partners, friends and families for cervical cancer patents in your community?   *Mungandiudze zvamunoziva pamusoro pe rutsigiro runopiwa vanhu vane gomarara remuromo wechibereko, kubva kune varume vavo, shamwari dzavo pamwe ne mhuri dzavo munharaunda yenyu?*   - How do you think partner, friend or family support help cervical cancer patients?   *Munofunga kuti vanhu vanegomarara remuromo wechibereko vanowana rutsigiro rwupi kubva kuvarume vavo, shamwari dzavo pamwe ne mhuri dzavo*   - What roles are churches in Harare playing to support cervical cancer patients? Probe for social, emotional and spiritual support. What are the general perceptions of people in your community with regards to cervical cancer? Probe for attitudes, beliefs, misconceptions and fears?   *Machechi emu Harare arikuita zvipi mukupa rutsigiro kune vane gomarara remuromo wechibereko? Bvunza pamusoro perubatsiro runechekuita nemagariro, zvavanonzwa ne mweya. Bvunza pamusoro pe mafungiro akajairika munharaunda yenyu akanangana ne gomarara remuromo wechibereko. Bvunza pamusoro pemafungiro, zvavanotenda, zvavazinga zwisise ne zvavanotya.* |
| **Access to cervical cancer treatment and palliative care**   - Tell me about cervical cancer treatment and palliative services in health facilities in Harare? Probe for names of health facilities.   *Mungandiudze maererano nekurapwa uye rubatsiro runopiwa ne vanochengeta vane gomarara remuromo wechibereko mu Harare? Bvunza mazita ezvipatara/ kana makiriniki aya.*   - Can you tell me your opinions about access to treatment and palliative services in Harare? Probe for who has better access and why?   *Mungandiudze mafungiro enyu pamusoro pekurapwa ne rutsigiro runopiwa nevanochengeta vano rwara negomarara mu Harare?Ndeanani vanowana rubatsiro uru rurinani? Sei madaro?*   - Can you tell me what you know about how cervical cancer is treated in health facilities? Probe if there are other means through which cervical cancer may be treated apart from health facilities?   *Mungandiudze zvamunoziva pamusoro pekurapwa kunoitwa gomarara remuromo wechibereko rino rapwa sei mumakirniki/zvipatara? Bvunza kana pane dzimwe nzira dzingashandiswe kurapa gomarara romuromo wechiberekokunze kweku enda kuchipatara/kukiriniki?*   - Who do you think can best treat and manage cervical cancer and why do you think so?   *Ndiyani wamunofunga kuti anogona kurapa gomarara remuromo wechibereko zviri nani uye sei muchifuna kudaro?*   - What are your opinions about service fees for cervical cancer treatment in Harare? Probe for affordability to patients or their families?   *Mungandi udze zvamunofunga pamusoro pe mari dzinodiwa kuti munhu arapwe gomarara remuromo wechibereko muHarare? Bvunza pamusoro*  *pekukwanisa kubhadara kwevarwere ne mhuridzavo?*   - What do you think about the availability of treatment services in Harare are adequate to cover all cervical cancer patients? Probe for reasons of response?   *Munofunga kuti zvipatara /makiriniki anowanikwa muHarare zvinorapa vanhu vanoda rubatsiro rwakadai zvakakwana? Bvunza zvekonzero zvaita kuti ape mhinduro yakadai.*   - What do you think about the adequacy of doctors and specialists to treat cervical cancer in Harare? Probe for reasons of response?   *Munofunga kuti mu Harare anachiremba na anamazvikokota vanorapa gomarara remuromo wechiberoko vanokwana? Bvunza pamusoro pemhinduro yawapiwa?*   - Do you think about the training of doctors and specialists to provide good treatment services to cervical cancer patients?   *Munofunga kuti anachiremba na anamazvikokota vakadzidziswa zvakakwana kuti varape vanhu zvakanaka?*   - What do you know about palliative care in health facilities in Harare? Probe for names of some of the facilities?   *Zvi zvamunofunga pamusoro pe nzvimbo dzinochengeta/ kubatsira vane gomarara remuromo wechibereko mu Harare?*   - Tell me what you know/think about the adequacy of palliative services to cover the patients who need such services?   *Munofunga kuti zvipatara /makiriniki anorapa vanhu vanoda rubatsiro rwakadai zvakakwana?*   - Describe some the challenges that patients in your community have experienced [or experience] in trying to access cervical cancer treatment or palliative care?   *Mungatsanangure kuti ndeapi matambudziko anosangana neavo vane gomarara remuromo wechibereko kana vachida kuno rapwa kana kuti kunowana rubatsiro kune avo vano chengeta vanegomarara mu munharaudna yenyu?* |
| **Utilization of cervical cancer treatment and palliative care**   - Can you tell me more about your understanding of health seeking behaviors by women in your community for treatment and palliative services for cervical cancer in Harare?   *Mungandiudze zvizere pamusoro pemazva munonzwisisa pamusoro penzira dzinotsvagwa nayo rubatsiro ne vanhukadzi vemunharaunda yenyu zvakanangana nekurapwa kana kuwana rutsigiro mu Harare zvine chekuita negomarara remuromo wechibereko .*   - In your opinion do think your community understands well treatment and palliative services offered for cervical cancer in health facilities?   *Munofunga kuti vanhu vemunharaunda yenyu vanonzwisisa zvakakwana pamusoro kurapwa nekubatsirwa kurukitwa avo vane gomarara remuromo wechibereko kunoshanda mumakiriniki/ zvipatara?*   - To your knowledge, tell me more about the treatment and palliative services being accessed by women in your community?   *Mukuziva kwenyu, mungandi udze zvizere pamusoro pekurapwa and kuchengetwa/kubatsirwa kuno itwa vanhukadzi veraimunharaunda yenyu?*   - What is your opinion of the effectiveness of treatment and palliative services provided in health facilities in Harare?   *Munofunga kuti kurapwa kurukitwa avo vane gomarara remuromo wechibereko mumakiriniki kana zvipatara e/zve muHarare kunoshanda here?*   - What do you think are some of the challenges that cervical cancer patients and their families are facing in using treatment and palliative care services in Harare?   *Munofunga kuti ndeapi matambudziko anosangana ne avo vanegomarara remuromo wechibereko kana vachida kuno rapwa kana kuti kunowana rubatsiro kune vano chengeta vanegomarara mu Harare?*  **Health services**   - Tell me about the information that health facilities provide about cervical cancer to your community?   *Ndeapi mashoko akanangana ne gomarara remuromo wechibereko amakawana kubva kukiriniki /kana chipatara emunharaunda yenyu.*   - According your understanding, what are the experiences of women when they notice signs and symptoms for cervical cancer? Probe about early health seeking behaviors?   *Mukunzwisisa kwenyu vanhukadzi vanosangana nezvipi kana vakaona kana kunzwa zvino ratidza kuti vane gomarara remuromo wechibereko? Bvunza pamusoro peku enda kunorapwa pachiri nenguva?*   - What are your opinions about adequacy of equipment, drugs, beds and other infrastructure to provide cervical treatment and palliative care in health facilities in Harare?   *Mukufunga kwenyu makiriniki/zvipatara e/zvemu Harare zvine zvakakwana zvakaita se mishini, mibhedha, mishonga ne zvimwe zvakadaro zvinoshandiswa mukurapa nekuchengeta vane gomarara remuromo wechibereko*   - What are your opinions about the adequacy of health workers (nurses, nurse aids, doctors, specialists, pharmacists, radiographers and laboratory scientists) to provide treatment and palliative care of cervical cancer patients in health facilities in Harare?   *Munofunga kuti makiriniki/zvipatara zvine vashandi vakakwana (anamukoti, anachiremba, anamazvikokota, vanopa mishonga nevano shanda mumalebhu) vakakwana uye vachikwanisa kurapa nekuchengeta vane gomarara remuromo wechibereko?*   - Can you tell me more about your opinions on the fees that are charged for treatment services by your health facility?   *Mungandiudze zvizere pamusoro pemafungiro enyu pamari dzinodiwa kuti vanhu varapwe mumakiriniki kana zvipatara?*   - To your knowledge what are the experiences of patient who cannot pay or do not afford to pay for treatment or other procedures at health facilities?   *Mukuziva kwenyu varhwere vasingakwanise kubhadara mari dzinodiwa kukiriniki /chipatara kuti varapwe vanosangana nei?*   - What are your perceptions on the quality of services provided at health facilities? Probe for attitudes of nurse aids, nurses, doctors, specialists, radiographers, pharmacists and other health workers?   *Mukufunga kwenyu mungati mhando yekurapwa/mashandirwo emuma kiriniki/zvipatara yakamirasei? Bvunza pamusoro pemapfungiro evakoti, anachiremba, anamazvikokota, nevanopa mishonga nevamwe vashandi vemuzvipatara?*   - In your opinion what are the perceptions and attitudes of your local leadership (herdmen, counsellors, and chiefs) on people using health services when they are sick or for routine checkups?   *Mukuwona kwenyu, akamira sei mafungiro evatungamiri venharaunda yako ( mahedhimeni, makhansela na namambo) zvakangana nekuenda kwevanhu kuzvipatara/makiriniki kana vachirwara kana kuti kuno ongororwa zvisinei kuti havazi kurwara?.*   - What do you think should be done at health facilities and generally in Zimbabwe to improve cervical cancer treatment and palliative care services?   *Ndezvipi zvamunofunga kuti zvinofanirwa kuitwa muzvipatara kana makiriniki kuvandudza kurapwa kwegomarara remuromo wechibereko ne kuchengetwa /kubatsirwa kwe vane gomarara iri mu Zimbabwe?* |

**Remarks:** Thank the participant for their time and proceed to the next one! **-------The End------------**
