# Supplementary material for: Model strategies to address barriers to cervical cancer treatment and palliative care among women in Zimbabwe: a public health approach
Source: BMC Womens Health. 2021 Apr 27;21:180. doi: 10.1186/s12905-021-01322-4 (PMC8077905; doi:10.1186/s12905-021-01322-4)
Supplement: Supplementary file 3 — Additional file 3. Focus discussion guide. [file 12905_2021_1322_MOESM3_ESM.docx]

**FOCUS DISCUSSION GUIDE**

| **Introductory questions**   - To get started, let’s introduce ourselves.   *Kuitira kuti titange ngatizivisanei kuti tirianani*   - Can each person say who they are: health woman, man or partner of cervical cancer patient/survivor or cervical cancer patient/survivor?   *Mumwe nemumwe nga ataure kuti akamirasei; Munhukadzi anehutano, murume kana mumwe we munhu anorwara ne gomarara remuromo wechibereko/ akararama shure kwekuva ne gomarara rechibereko*   - Can each person say where they stay?   *Tinokumbira muti udze kuti munogara kupi.* |
| --- |

| **Knowledge of cervical cancer**   - What have you heard about cervical cancer? Probe for causes and risk factors?   *Makambonzwa nezve gomarara remuromo we chibereko? Bvunza pamusoro pezvinokonzera gomarara iri.*   - Tell us how you would prevent cervical cancer? Probe for screening and treatment of precancerous lesions, vaccination and male circumcision of partners.   *Mungandiudze kuti mungadzivirire sei kuwavana gomarara remuromo wechibereko? Bvunza pamusoro pekuongororwa nekurapwa kwezvironda zvinovapo gomarara risati ratanga, kubayiwa mishonga yekudzivirira ne kuchecheudzwa kwevarume?*   - Tell us some of the signs and symptoms of cervical cancer?   *Mungandiudze kuti ndezvipi zvinoratidza nezvino nzwikwa nemhunu anegomarara remuromo wechibereko?*   - Do you think people in your community are at risk of developing this disease? Probe for reasons of responses.   *Munofunga kuti vanhu vemunharaunda yenyu varipanjodzi yekuva ne gomarara iri? Bvunza pamusoro pemhinduro yapihwa.* |
| --- |
| **Experiences of cervical cancer**   - What are some of the reasons why women go for cervical cancer screening? Probe for routine screening, health problems, health worker advice, friend or relative advice?   *Ndezvipi zvimwe zvikonzero zvinoita kuti vanhukadzi vaende kuno ongororwa gomarara remuromo wechibereko?Bvunza pamusoro pekuenda kuno ongororwa nguva ne nguva, matambudziko ehutano, kukurudzirwa nevanoshanda kuzvupatara, ne shamwari kana hama?*   - What are some of the experiences with cervical cancer patients that you can tell us more about?   *Ndezvipi zvamakasangana nazvo mukushana nevanhu vakararama shure mekuva ne gomarara remuromo wechibereko*?   - Do you know or have you ever heard about palliative care services? Probe for what they know or have heard about palliative care?   *Munofunga kuti vanhu vanoziva kana kuti vakambonzwa nezve kurapwa nerutsigirwo runopiwa neavo vanochengeta vanegomarara remuromo wechibereko? Zvii zvamunoziva pamusoro pe izvi?*   - Do you think cervical cancer patients receive support from their partners, friends and families?   *Munofunga kuti vanhu vane gomarara remuromo wechibereko vanowana rutsigiro kubva kuvarume vavo, shamwari dzavo pamwe ne mhuri dzavo*   - How do you think partners, friends or families support help cervical cancer patients?   *Munofunga kuti vanhu vanegomarara remuromo wechibereko vanowana rutsigiro rwupi kubva kuvarume vavo, shamwari dzavo pamwe ne mhuri dzavo*   - Are churches in Harare providing social, emotional and spiritual support to cervical cancer patients and their families? Probe for reasons for the answer given?   *Machechi emu Harare arikupa rutsigiro runechekuita nehunzwa ne mweya kuvanhu vane gomarara remuromo wechibereko pamwe ne mhuri dzavo? Bvunza pamusoro pemhinduro dzinenge dzapiwha.*   - What are the general attitudes of people in your community with regards to cervical cancer? Probe for perceptions, beliefs, misconceptions and fears?   *Ndeapi mafungiro akajairika munharaunda yenyu akanangana ne gomarara remuromo wechibereko. Bvunza pamusoro pemafungiro, zvavanotenda, zvavazinga zwisise ne zvavanotya*? |
| **Access to cervical cancer treatment and palliative care**   - Which health facilities that you know or have heard about provide cervical cancer treatment and palliative care in Harare?   *Ndekupi muHarare kwamunoziva kana kwamakanzwa kuti kuno rapwa gomarara remuromo wechibereko kana kuno wanikwa rubatsiro kune vanochengeta vane gomarara?*   - Do you think members of your community have access to cervical cancer diagnosis, treatment and palliative care services? Who have better access and why is that so?   *Munofunga kuti vanhu vemunharaunda yenyu vanokwanisa kuwana rubatsiro nekuongororwa uye nerutsigiro kune vanochengeta vano rwara negomarara?Ndeanani vanowana rubatsiro uru? Sei madaro?*   - How is cervical cancer treated in health facilities? Probe if there are other means through which cervical cancer may be treated?   *Gomarara remuromo wechibereko rino rapwa sei mumakirniki/zvipatara? Bvunza kana pane dzimwe nzira dzingashandiswe kurapa gomarara romuromo wechibereko?*   - Who do you think can best treat and manage cervical cancer?   *Ndiyani wamunofunga kuti anogona kurapa gomarara remuromo wechibereko zviri nani?*   - Do you think cervical cancer treatment and palliative services are affordable to you or members of your community?   *Munofunga kuti kurapwa kana kuwana rubatsiro rune chekuita nekuchengetwa kwe vane gomarara remuromo wechibereko kwakachipa kwamuri kana vanhu venunharaunda yenyu?*   - Do you think the treatment services available in Harare are adequate to cover all cervical cancer patients?   *Munofunga kuti zvipatara /makiriniki anowanikwa muHarare zvinorapa vanhu vanoda rubatsiro rwakadai zvakakwana?*   - Do you think there are sufficient doctors and specialists to treat cervical cancer in Harare?   *Munofunga kuti mu Harare anachiremba na anamazvikokota vanorapa gomarara remuromo wechiberoko vanokwana? Bvunza pamusoro pemhinduro yawapiwa?*   - Do you think that the available doctors and specialists are adequately trained to provide good treatment services?   *Munofunga kuti anachiremba na anamazvikokota vakadzidziswa zvakakwana kuti varape vanhu zvakanaka?*   - Do you think these facilities are adequate to cover the patients who need such services?   *Munofunga kuti zvipatara /makiriniki anorapa vanhu vanoda rubatsiro rwakadai zvakakwana?*   - Describe some of the challenges that you/partner or other member of your family or community have experienced [experience] in trying to access cervical cancer treatment or palliative care?   *Mungatsanangure kuti ndeapi matambudziko anosangana neavo vane gomarara remuromo wechibereko kana vachida kuno rapwa kana kuti kunowana rubatsiro kune avo vano chengeta vanegomarara mu munharaudna yenyu?* |
| **Utilization of cervical cancer treatment and palliative care**   - If you or partner or relative were diagnosed of cervical cancer where would you take or encourage them to go for treatment? Why?   *Imi kana kuti mumwewenyu kana hama yenyu ikabatwa gomarara remuromo wechibereko mungaende kana kukurudzira kuti vaende kunorapwa? Bvunza zvikonzero?*   - Do you think that most women with cervical cancer are being treated? Who are those getting treatment and palliative care? Why?   *Munofunga kuti vanhukadzi vazhinji variku rapwa gomarara rechibereko? Ndevapi vari kurapwa ne varikuwana rubatsiro kubva mune vanochengeta vane gomarara iri?*   - Where do you think most women are being treated for cervical cancer in Harare?   *Muno muHarare munofunga kuti vanhukadzi vazhinji vanono rapwa gomarara rechibereko kupi?*   - In your opinion do you think treatments given to cervical cancer patients at health facilities are effective? Probe for reasons?   *Munofunga kuti kurapwa kurukitwa avo vane gomarara remuromo wechibereko kunoshanda here? Bvunza pamusoro pezvikonzero?*   - What do you think are some of the challenges that cervical cancer patients and their families are facing in using treatment and palliative care services in Harare?   *Munofunga kuti ndeapi matambudziko anosangana ne avo vanegomarara remuromo wechibereko kana vachida kuno rapwa kana kuti kunowana rubatsiro kune vano chengeta vanegomarara mu Harare?*  **Health services**   - What information have you received from your health facility with regards to cervical cancer?   *Ndeapi mashoko akanangana ne gomarara remuromo wechibereko amakawana kubva kukiriniki /kana chipatara*   - If you or a member of your household were to have signs and symptoms of cervical cancer, what would you do? Why?   *Kana munhu wemunharaunda ino akava nezvino ratidza kana kunzwa zvino nzwikwa nevane gomarar remuromo we chibereko, angaite sei? Sei wadaro?*   - Do you think people in your community seek treatment early when they suspect cervical cancer?   *Munofunga kuti vanhu vemunharaunda yenyu vanotsvaga rubatsiro nguva ichiripo kana vachifungidzira kuti vane gomarara remuromo wechibereko?*   - Do you think health facilities treating cervical cancer have adequate equipment, drugs, beds and other infrastructure to provide cervical treatment and palliative care?   *Munofunga kuti makiriniki/zvipatara zvine zvakakwana zvakaita se mishini, mibhedha, mishonga ne zvimwe zvakadaro zvinoshandiswa mukurapa nekuchengeta vane gomarara remuromo wechibereko*   - Do you think health facilities have adequate health workers (nurses, nurse aids, doctors, specialists, pharmacists, radiographers and laboratory scientists) to provide treatment and palliative care of cervical cancer patients?   *Munofunga kuti makiriniki/zvipatara zvine vashandi vakakwana (anamukoti, anachiremba, anamazvikokota, vanopa mishonga nevano shanda mumalebhu) vakakwana uye vachikwanisa kurapa nekuchengeta vane gomarara remuromo wechibereko?*   - In your opinion are the service fees for cervical cancer treatment affordable to patients or their families?   *Mukufunga kwenyu varhwere nemhuri dzavo vanokwanisa kubhadara mari dzinodiwa kukiriniki /chipatara kuti varapwe.*   - Let us imagine that a patient does not have funds to pay for treatment or other procedures would they still access services?   *Tichifungidzira, munhu asina mari yekubhdara kuti arapwe gomarara anokwanisa kurapwa here?*   - What are your perceptions on the quality of services provided at health facilities? Probe for attitudes of nurse aids, nurses, doctors, specialists, radiographers, pharmacists and other health workers?   *Mukufunga kwenyu mungati mhando yekurapwa/mashandirwo emuma kiriniki/zvipatara yakamirasei? Bvunza pamusoro pemapfungiro evakoti, anachiremba, anamazvikokota, nevanopa mishonga nevamwe vashandi vemuzvipatara?*   - Does your local leadership (herdmen, counsellors, and chiefs) encourage people to use health services when they are sick or for routine checkups?   *Vatungamiri venharaunda yako ( mahedhimeni, makhansela na namambo) vanokurudzira kuti vanhu vaende kuzvipatara/makiriniki kana vachirwara kana kuti kuno ongororwa zvisinei kuti havazi kurwara?.*   - What do you think should be done at health facilities and generally in Zimbabwe to improve cervical cancer treatment and palliative care services?   *Ndezvipi zvamunofunga kuti zvinofanirwa kuitwa muzvipatara kana makiriniki kuvandudza kurapwa kwegomarara remuromo wechibereko ne kuchengetwa /kubatsirwa kwe vane gomarara iri mu Zimbabwe ?* |

**Remarks:** Thank the participants for their time.

**-----------------------------------------------------The End---------------------------------------------------------**
